# Soil bacterial community is more sensitive than fungal community to canopy nitrogen deposition and understory removal in a Chinese fir plantation

**DOI:** 10.3389/fmicb.2022.1015936

**Published:** 2022-10-12

**Authors:** Dan Xi, Shaofei Jin, Jianping Wu

**Affiliations:** ^1^Lushan Botanical Garden, Chinese Academy of Sciences, Jiujiang, China; ^2^College of Forestry, Fujian Agriculture and Forestry University, Fuzhou, China; ^3^Department of Geography, Minjiang University, Fuzhou, China; ^4^Yunnan Key Laboratory of Plant Reproductive Adaptation and Evolutionary Ecology, Institute of Biodiversity, Yunnan University, Kunming, China; ^5^Key Laboratory of Soil Ecology and Health in Universities of Yunnan Province, School of Ecology and Environmental Sciences, Yunnan University, Kunming, China

**Keywords:** canopy N deposition, understory removal, microbial community, bacterial diversity, Chinese fir plantation

## Abstract

Soil microorganisms are key regulators for plant growth and ecosystem health of forest ecosystem. Although previous research has demonstrated that soil microorganisms are greatly affected by understory nitrogen (N) addition, little is known about the effects of canopy N addition (CNA) and understory management on soil microorganisms in forests. In this study, we conducted a full designed field experiment with four treatments: CNA (25 kg N ha^–1^ year^–1^), understory removal (UR), canopy N addition, and understory removal (CNAUR) (25 kg N ha^–1^ year^–1^), and control in a Chinese fir plantation. High-throughput sequencing and qPCR techniques were used to determine the abundance, diversity, and composition of bacterial and fungal communities in three soil layers. Our results showed that CNA increased bacterial diversity in the 10–20 cm soil layer but decreased bacterial abundance in the 20–40 cm soil layer and fungal diversity in the 0–10 cm soil layer. UR increased bacterial abundance only in the 20–40 cm soil layer. CNA, not UR significantly altered the compositions of soil bacterial and fungal community compositions, especially in the 0–20 cm soil layer. CNA sharply reduced the relative abundance of copiotrophic taxa (i.e., taxa in the bacterial phylum Proteobacteria and the orders Eurotiales and Helotiales in the fungal phylum Ascomycota) but increased the relative abundance of oligotrophic taxa (i.e., in the bacterial phylum Verrucomicrobia). RDA analysis revealed that soil pH, DON, and DOC were the main factors associated with the variation in bacterial and fungal communities. Our findings suggest that short-term CNA changes both soil bacterial and fungal communities, with stronger responses in the surface and middle soil than in the deep soil layer, and that UR may enhance this effect on the soil bacterial abundance. This study improves our understanding of soil microorganisms in plantations managed with understory removal and that experience increases in N deposition.

## Introduction

Deposition of active nitrogen (N) has dramatically accelerated because of anthropogenic activities (Galloway et al., 2004; Yu et al., 2019), and the effects of N deposition on terrestrial ecosystems have been widely investigated (Chen et al., 2015; Wu et al., 2019; Lu X. K. et al., 2021; Peguero et al., 2021). Most of the previous studies on the effects of N deposition on forests involved the experimental addition of N fertilizer to the forest floor (e.g., Krumins et al., 2009; Wu et al., 2019; Lu X. K. et al., 2021; Peguero et al., 2021) or a natural N-deposition gradient (Zechmeister-Boltenstern et al., 2011). With natural N deposition in forests, the quantity of N reaching the floor can be reduced by an interception with the canopy and the understory. In studies of N deposition in forests, such interception and retention have largely been ignored (Liu et al., 2020; Tian et al., 2020), likely resulting in overestimates of the effects of N deposition on forest ecosystems. Moreover, recent studies have indicated that forest canopies can intercept 30 to 85% of N deposition (Houle et al., 2015; Liu N. et al., 2018; Liu et al., 2020; Wang X. et al., 2021) and that elevated N deposition does not adversely affect microorganisms (Liu et al., 2020), fauna (Tian et al., 2020) or carbon (C) processing in the soil (Lu X. F. et al., 2021). Previous studies have also reported that canopy N addition (CNA) increased the species richness of the shrub layer (Tian et al., 2019) and increased the size of xylem tracheids of dominant broadleaf species (Jiang et al., 2018). These studies suggest the effects of N addition differ depending on where the N is added (i.e., directly to the forest floor or to the canopy). Overall, our understanding of how CNA affects forest ecosystems is incomplete.

Soil microorganisms are important regulators of nutrient cycling and litter decomposition processes (Zeng et al., 2016) that greatly affect plant growth and ecosystem health (Wall et al., 2015). It is well known that soil microorganisms are sensitive to environmental change and that increases in N deposition are likely to change soil microbial communities by altering nutrient availability (Ma et al., 2020). Many studies have reported that N deposition significantly affects soil microbial biomass, diversity, and community composition in subtropical forests (Mo et al., 2008; Liu et al., 2015; Li et al., 2016; Wang et al., 2018; Tian et al., 2019; Wu et al., 2019) and in temperate and boreal forests (Boot et al., 2016). In contrast, a recent study found that the soil microbial community was not influenced by N addition in a tropical rainforest (Li P. et al., 2019). As noted earlier, however, most studies of the response of soil microorganisms to increasing N deposition in forest ecosystems have involved N addition to the forest floor (e.g., Mo et al., 2008; Li et al., 2016; Wu et al., 2019; Wang J. P. et al., 2021), and only three studies have determined how the soil microbial community responds to CNA (Shi et al., 2016, 2018; Zhao et al., 2020). Research is needed to clarify how soil microbial communities in forests respond to CNA. In addition to being affected by how N is added, the response of soil microorganisms to N addition can be affected by forest or stand type (Zechmeister-Boltenstern et al., 2011; Zhou et al., 2017). Shi et al. (2016) reported that the effect of CNA on soil microbial biomass differed between subtropical and temperate forests. Fan and Hong (2001) found that the canopies of coniferous species, like those of broadleaf species, also intercepted substantial proportions of N before N reached the soil. Whether the response of the soil microbial community to CNA in conifer forests is similar to that in broadleaf forests has yet to be investigated.

Understory vegetation removal is an important silviculture practice in plantation ecosystems (Wang T. et al., 2021). The understory is removed to reduce competition between tree and understory species and increase lumber production (Wu et al., 2011; Wang et al., 2014). On the other hand, removing understory vegetation also alters soil temperature and moisture (Xiong et al., 2008; Wang et al., 2014; Giuggiola et al., 2018) and nutrient availability, which greatly affect soil microorganisms. Several studies reported that understory removed reduced soil microbial biomass due to the increased soil temperature or reduced substrate availability in subtropical forests (Xiong et al., 2008; Wu et al., 2011; Zhao et al., 2013; Wan et al., 2019), and one study found that soil microbial properties were not significantly affected by understory removal (Urcelay et al., 2010). It, therefore, remains unclear how understory removal influences the soil microbial community. By increasing N availability, N addition was previously found to enhance forest understory regeneration (Trentini et al., 2018). Removal of understory plants is likely to increase the proportion of N deposition that reaches the floor and thereby increase soil N availability, which is likely to stimulate or suppress microbial activity and consequently result in changes in microbial biomass, diversity, and community composition. Previous phospholipid fatty acids (PLFAs) analyses indicated a reduction in fungal biomass after understory N addition and understory removal in subtropical plantations (Zhao et al., 2013; Lei et al., 2018). However, these previous studies did not indicate which specific microbial groups were affected by N addition and understory removal.

Plantations of Chinese fir (*Cunninghamia lanceolata*) have been widely planted in the subtropical regions of China due to the tree’s ecological and economic value (Li R. S. et al., 2019). As previously indicated, understory plants are usually removed from these plantations to increase timber quality and production (Li R. S. et al., 2019), and these subtropical regions have higher N deposition than other regions in China (Yu et al., 2019). Although the effects of N addition or understory removal on soil microorganisms have been studied in plantations, the data are mostly limited to the surface/subsurface soil layer (<20 cm) (Wu et al., 2011, 2019; Lei et al., 2018; Li R. S. et al., 2019) even though nutrient levels are known to change with soil depth (Liu D. et al., 2018; Kang et al., 2021). It remains unclear how the soil microbial community at different soil depths is affected by the interaction of N deposition and understory removal in Chinese fir plantations. A better understanding of the effects of N deposition, understory removal, and soil depth on soil fungi and bacteria will help clarify the mechanisms affecting nutrient availability in these plantations. Understanding the changes in microbial diversity and community structure in these soil layers could also contribute to forest management.

This study performed a 5-year CNA and understory removal experiment on a Chinese fir plantation. The objectives were to determine whether CNA, understory removal, or their interaction affected the abundance, diversity, and structure of soil bacterial and fungal communities in different soil layers and identify which factors were associated with these effects. We tested three hypotheses: (1) short-term CNA would not significantly affect microbial communities in surface or deeper soil layers due to the substantial interception of N by the canopy (Shi et al., 2016); (2) by reducing the belowground input of C, understory removal would affect the bacterial community more than the fungal community because nutrient demand and metabolic activities are lower for fungi than bacteria (Zhou et al., 2017); and (3) changes in the composition of soil microbial communities will be associated with different soil properties in different soil layers.

## Materials and methods

### Site description

This study was carried out at the Guanzhuang National Forestry Farm (117°43′ E, 26°30′ N) in Sanming City, Fujian Province, China. The region has a subtropical monsoon climate. The average annual temperature and precipitation are 18.8–19.6°C and 1,606–1,650 mm, respectively, and the soil is classified as acrisol (Wu et al., 2019).

The Chinese fir plantation used in this study was established in 2008 on a 4-ha site with homogenous conditions. At the start of the experiment in June 2013, the dominant understory plants were *Bambusa chungii* and *Dicranopteris dichotoma* (accompanied by *Smilax china* and *Melastoma dodecandrum*); the mean fir tree height and diameter at breast height were 5.11 m and 7.34 cm, respectively; the average contents of total organic carbon (TOC) and total nitrogen (TN) in the soil surface layer were 33.37 and 1.91 kg^–1^, respectively; and the mean pH was 4.44 (Lei et al., 2018).

### Experimental design

Eight plots (15 m × 15 m for each plot), four plots with N addition treatments and four plots without N addition, were randomly selected. A 5-m × 10-m understory removal subplot was established in each plot. As a result, the experiment included two levels of N addition (±) and two levels of understory removal (±) in a factorial design that resulted in four treatments: no N addition and no understory removal (control, CK); understory removal without N addition (UR, 0 kg N ha^–1^ year^–1^); CNA without understory removal (CNA, 25 kg N ha^–1^ year^–1^); and canopy N addition plus understory removal (CNAUR) (25 kg N ha^–1^ year^–1^). Adjacent plots were separated by a 3- to 8-m wide buffer strip to prevent cross-contamination. To add N to a plot, the required quantity of NH_4_NO_3_ (269 g) was dissolved in 15 L of water, and the solution was sprayed onto the tree canopy using a previously described spraying system (Lei et al., 2018). Control plots received an equivalent volume of water without NH_4_NO_3_. Plots were treated once every 2 months starting in June 2014 and continuing until sampling in April 2019. All understory plants were manually removed in UR and CNAUR plots at the start of the experiment, and germinating understory plants in UR and CNAUR plots were manually removed every 2 months.

### Sample collection and analyses

In April 2019, eight soil cores (3.5 cm diameter) were randomly collected at depths of 0–10, 10–20, and 20–40 cm in each plot and were combined to yield one composite sample per depth per plot. Litter was removed from the soil surface before the cores were taken. The fresh soil samples were passed through a 2-mm sieve. Visible roots and stones were carefully removed before each composite soil sample was divided into three parts. One part was used for the determination of soil moisture and contents of soil ammonium (NH_4_^+^), nitrate (NO_3_^–^), microbial biomass carbon (MBC) and microbial biomass nitrogen (MBN), and dissolved organic carbon (DOC) and dissolved organic nitrogen (DON). Another part was air-dried to the determination of soil pH and contents of TOC, TN, and available phosphorus (AP). The third part was stored at −80°C for microbial molecular analysis.

Soil TOC and TN contents were measured with an element analyzer (Vario isotope cube, Germany). Soil NH_4_^+^ and NO_3_^–^ contents were determined with a chemical analyzer (SmartChem 200, Italy). Soil MBC and MBN were analyzed using a chloroform fumigation-extraction method. Soil DOC and TDN were extracted with a 0.5 M K_2_SO_4_ solution and were measured with a total organic analyzer (TOC-V CPH, Japan); DON was calculated by subtracting inorganic N (NH_4_^+^ and NO_3_^–^) from TDN. Soil pH was measured in a 1:2.5 (w/v) mixture of soil and deionized water with a pH meter. Soil moisture was determined by the gravimetrical weight method by drying fresh soil at 105°C to a constant weight. Soil AP was extracted with a 0.05 M NH_4_F–0.01 M HCl solution and was determined with the colorimetrical molybdate blue method.

### DNA extraction, PCR amplification, and sequence processing

Microbial DNA was extracted from 0.5-g subsamples of fresh soil samples using the PowerSoil DNA Isolation Kit (MO Bio Laboratories Inc., Carlsbad, CA, USA) according to the manufacturer’s protocols. The final DNA concentration and purification were determined with a NanoDrop 2000 UV-vis spectrophotometer (Thermo Scientific, Wilmington, DE, USA), and DNA quality was checked by 1% agarose gel electrophoresis.

The V3–V4 hypervariable region of the bacterial 16S rRNA gene was amplified using paired primers 338F (5′-ACTCCTACGGGAGGCAGCA-3′) and 806R (5′-GGACTACHVGGGTWT CTAAT-3′) by a PCR thermal cycler (GeneAmp 9700, ABI, Cambridge, MA, USA). The PCR amplification procedure for bacteria was as follows: 95°C for 3 min; followed by 29 cycles of 30 s at 95°C for denaturation, 30 s at 55°C for annealing, and 45 s at 72°C for extension; and a final extension at 72°C for 10 min. The rDNA internal transcribed spacer (ITS) region 1 of fungi was amplified using the primers ITS1F (5′-CTTGGTCATTTAGAGGAAGTAA-3′) and ITS2R (5′-GCTGCGTTCTTCATCGATGC-3′). The PCR amplification procedure was as follows: initial denaturation at 95°C for 3 min; followed by 37 cycles at 95°C for 30 s, 55°C for 30 s, and 72°C for 45 s; and a final extension at 72°C for 10 min. The PCR products of both bacteria and fungi were further purified, quantified, and finally sent for sequencing on an Illumina MiSeq PE300 platform at Majorbio Bio-Pharm Technology Co., Ltd., Shanghai, China. The bacterial and fungal gene sequences were deposited in NCBI under accession number SRP264464.

High-quality sequences were obtained by filtering the raw reads with the three criteria reported by Wu et al. (2019). Sequences with >97% similarity were clustered to the same operational taxonomic units (OTUs) using UPARSE software (version 7.1) (Edgar, 2013). The taxonomy of each bacterial and fungal OTU was determined by the RDP classifier (version 2.11),^1^ and the selected representative sequences were implemented in the SILVA database (Release138)^2^ and the UNITE database (Release 8.0)^3^ for identification of bacteria and fungi, respectively. The Chao 1 index, the Shannon Index, and the number of observed OTUs were calculated to assess the alpha-diversity of the bacterial and fungal community in each sample.

### Quantitative PCR for bacteria and fungi

qPCR was used to determine the absolute abundance of bacterial 16S rRNA and fungal ITS genes. The target primers for bacteria and fungi were the same primers used for the PCR amplification process described in the previous section. The PCR reactions were carried out using an ABI Real-time 7500 system (Applied Biosystems, Waltham, MA, USA) and SYBR Green Premix (TaKaRa Bio Inc., Shiga, Japan). The PCR protocol for both bacteria and fungi involved an initial denaturation at 94°C for 5 min; followed by 30 cycles at 94°C for 30 s, 55°C for 30 s, and 72°C for 30 s; and a final extension at 72°C for 5 min. All qPCR reactions in each DNA sample were run in triplicate. Ten-fold serial dilutions of a plasmid containing the full-length target genes were used to generate the standard curves for quantitative PCR. The 16S rRNA and ITS gene abundances in all samples were expressed as copies per gram of dried soil.

### Statistical analysis

Statistical analyses were carried out with the SPSS 16.0 software package for Windows (SPSS Inc., Chicago, IL, USA). One-way ANOVAs were used to determine differences in physicochemical properties, microbial biomass (C and N), gene abundance, diversity indexes, and dominant community species among treatments in each soil layer; when ANOVAs were significant, means were compared by the LSD method. Two-way ANOVAs were used to assess the effects of CNA, understory removal on soil physicochemical properties and microbial communities in each soil layer. Pearson correlation analysis was used to examine the relationships between microbial abundance and diversity with soil properties, and a heatmap of microbial community composition at the order level and soil properties was generated using the “corrplot” package in R (version 4.0.5). Differences in the structure of bacterial and fungal communities among treatments were examined using principal coordinate analysis (PCoA) with non-parametric multivariate statistical methods (ADONIS). The relationships between microbial community properties and soil properties in each soil layer were assessed by redundancy analysis (RDA) with the Mantel test in the “vegan” package of R software (version 4.0.5). The structure equation model (SEM) was performed in the “piecewiseSEM” package (Lefcheck, 2016). All effects at *p* < 0.05 were considered statistically significant, and all analyses were performed using R software (R version 4.0.5).

## Results

### Soil physicochemical properties

The following properties within each soil layer were unaffected by the treatments: pH, soil moisture, TOC, TN, NH_4_^+^, NO_3_^–^, and DOC (Table 1). Soil AP content in the 0–10 cm layer was lower for the CNA treatment than for the CK treatment (Table 1). A similar pattern was found for DON content in the 20–40 cm layer. Two-way ANOVAs showed that CNA and UR did not influence soil physicochemical properties in different soil layers, whereas their statistical interactions (CNA × UR) had a marginal effect on soil AP and DON (Supplementary Table 1).

**TABLE 1:** Soil properties under different treatments.

Soil layer (cm)	Treatment	pH	Soil moisture (%)	TOC (g kg^–1^)	TN (g kg^–1^)	NH_4_^+^-N (mg kg^–1^)	NO_3_^–^-N (mg kg^–1^)	DON (mg kg^–1^)	DOC (mg kg^–1^)	AP (mg kg^–1^)
0–10	CK	4.33 (0.04) a	24.41 (0.87) a	23.67 (2.04) a	1.29 (0.08) a	11.80 (2.54) a	1.89 (0.44) a	16.77 (1.22) a	560.9 (65.4) a	10.34 (1.74) a
	UR	4.36 (0.08) a	24.12 (0.96) a	24.38 (2.96) a	1.31 (0.15) a	8.24 (1.27) a	1.61 (0.31) a	16.26 (0.85) a	551.3 (44.4) a	7.10 (1.07) ab
	CNA	4.38 (0.04) a	24.18 (0.86) a	20.37 (1.75) a	1.05 (0.10) a	7.36 (0.98) a	1.21 (0.32) a	17.16 (1.36) a	531.2 (52.6) a	6.49 (0.15) b
	CNAUR	4.41 (0.04) a	24.87 (1.06) a	27.27 (3.24) a	1.47 (0.22) a	9.05 (1.82) a	1.26 (0.08) a	17.46 (4.22) a	520.7 (81.8) a	8.60 (1.41) ab
10–20	CK	4.39 (0.02) a	22.73 (0.45) a	14.86 (2.03) a	0.91 (0.11) a	4.56 (0.06) a	1.58 (0.45) a	19.47 (1.76) a	587.1 (53.2) a	5.20 (0.95) a
	UR	4.33 (0.02) a	22.38 (1.16) a	14.92 (1.45) a	0.93 (0.09) a	5.31 (0.56) a	1.18 (0.21) a	17.52 (1.48) a	578.0 (53.5) a	3.47 (0.54) a
	CNA	4.37 (0.04) a	21.34 (0.94) a	12.47 (1.17) a	0.78 (0.08) a	4.43 (0.91) a	1.16 (0.15) a	16.01 (1.10) a	544.1 (51.5) a	3.36 (0.26) a
	CNAUR	4.36 (0.02) a	22.13 (0.68) a	13.67 (1.21) a	0.83 (0.09) a	5.08 (0.81) a	0.88 (0.16) a	16.98 (1.64) a	593.2 (63.1) a	3.80 (0.78) a
20–40	CK	4.32 (0.06) a	19.09 (0.45) a	8.24 (0.74) a	0.55 (0.05) a	2.91 (0.44) a	1.58 (0.19) a	14.39 (1.35) a	498.1 (81.8) a	2.42 (0.70) a
	UR	4.34 (0.01) a	20.59 (1.57) a	7.90 (1.04) a	0.57 (0.09) a	3.14 (0.39) a	1.44 (0.32) a	10.93 (1.20) ab	510.2 (63.9) a	2.32 (0.41) a
	CNA	4.34 (0.01) a	19.29 (0.76) a	8.18 (0.99) a	0.54 (0.04) a	3.72 (1.73) a	1.74 (0.59) a	10.20 (0.55) b	453.1 (71.9) a	1.36 (0.17) a
	CNAUR	4.18 (0.13) a	17.51 (1.13) a	8.29 (1.39) a	0.57 (0.09) a	2.69 (0.50) a	0.72 (0.10) a	11.68 (1.36) ab	427.7 (69.5) a	3.36 (1.71) a

Data are presented as mean and standard errors (in brackets) for replicates (n = 4). Different letters within columns indicate significant differences among treatments in the same soil layer.

### Gene abundance and microbial biomass carbon and nitrogen

**FIGURE 1:**
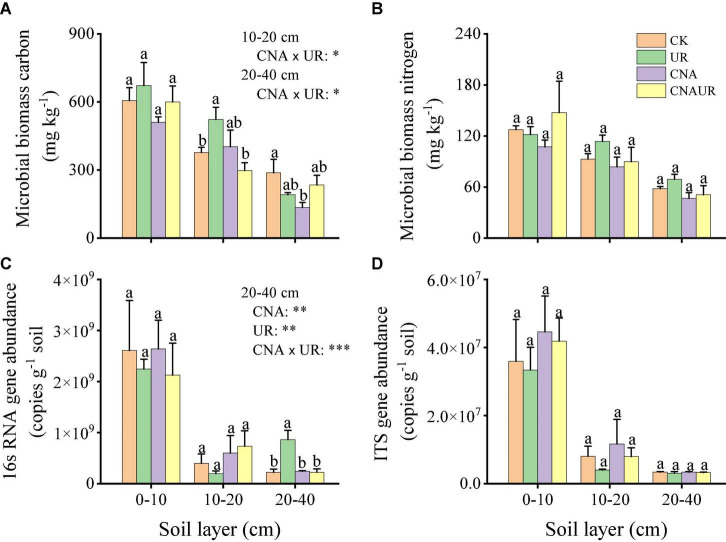
Contents of microbial biomass C **(A)** and N **(B)** and gene abundances of bacteria **(C)** and fungi **(D)** in three soil layers as affected by four treatments, i.e., two levels (±) of canopy N addition (CNA) and two levels (±) of understory removal (UR), and the statistical interaction CNA × UR. Treatments are described in Table 1. Values are means + SE. In each plot, means with different letters are significantly different at *p* < 0.05. *, **, and *** indicate significance at *p* < 0.05, 0.01, and 0.001, respectively.

**FIGURE 2:**
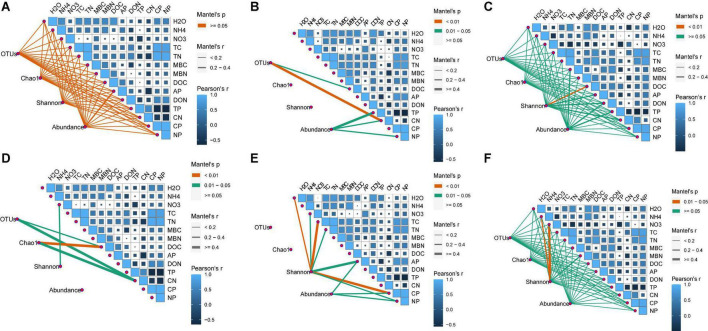
Correlations among soil chemical parameters and bacterial **(A–C)** and fugal **(D–F)** diversity indexes (OTUs, Chao1, Shannon, and gene abundance) in different soil layers, 0–10 cm **(A,D)**, 10–20 cm **(B,E)**, and 20–40 cm **(C,F)**. The correlation coefficients of each soil chemical parameter are presented by area size of squares, smaller area lower correlation, and vice versa. The level of correlations between microbial index and soil chemical variables is indicated by line thickness; the correlations are indicated by the line color; red and blue colors indicate positive and negative correlation, respectively.

Abundance was higher for the bacterial 16S rRNA gene (range = 1.97 × 10^8^ to 2.61 × 10^9^ copies g^–1^ soil) than for the fungal ITS gene (range = 4.01 × 10^6^ to 4.46 × 10^7^ copies g^–1^ soil) (Figures 1C,D). In the 0–20 cm soil layer, microbial gene abundances were not significantly affected by the treatments. However, in the 20–40 cm soil layer, bacterial gene abundance was significantly higher for the UR treatment than for the CK, UN, or CNAUR treatments (Figure 1C). Moreover, two-way ANOVAs demonstrated that bacterial gene abundance in this soil layer was significantly affected by CNA, UR, or their interaction (Figure 1C; Supplementary Table 2). In addition, bacterial gene abundance was negatively correlated with MBC and MBN in the 10–20 cm soil layer (Figure 2).

Soil MBC but not MBN was significantly affected by the CNA and UR treatments (Figures 1A,B). In the 10–20 cm layer, soil MBC content was significantly higher for the UR treatment than for the CK or the CNAUR treatment. In the 20–40 cm layer, soil MBC content was significantly lower for the CNA treatment than for the CK treatment. The statistical interaction between CNA and UR was significant for soil MBC in the 10–40 cm layer (Figure 1A; Supplementary Table 2).

### Soil bacterial and fungal diversity

**FIGURE 3:**
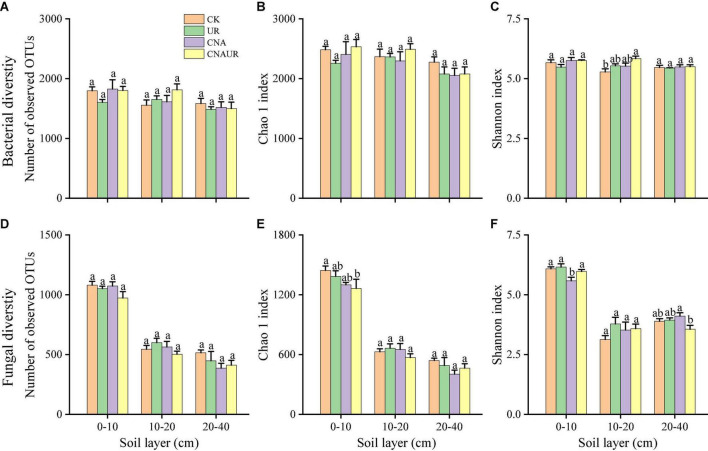
Diversity indices of bacteria **(A–C)** and fungi **(D–F)** in three soil layers as affected by four treatments (treatments are described in Table 1). Values are means ± SE. In each plot, means with different letters are significantly different at *p* < 0.05.

The number of OTUs and the Chao1 and Shannon indices were used to assess microbial diversity. For bacteria, the number of OTUs and the Chao1 index did not significantly differ among the treatments in any soil layer (Figures 3A,B). However, the Shannon index for bacteria in the 10–20 cm soil layer was significantly higher for the CNAUR treatment than for the CK treatment (Figure 3C). For fungi, the number of OTUs did not significantly differ among the treatments in any soil layer (Figure 3D). The Chao1 index for fungi was significantly higher in the CK treatment than in the CNAUR treatment in the 0–10 cm soil layer (Figure 3E). The Shannon index for fungi was significantly lower in the CNA treatment than in the other treatments in the 0–10 cm soil layer; it did not differ among the treatments in the 10–20 cm soil layer and was significantly higher in the CNA treatment than in the CNAUR treatment in the 20–40 cm soil layer (Figure 3F). Overall, the effects of CNA and UR on bacterial and fungal diversity differed among the soil layers (Figure 3; Supplementary Table 2). In addition, the Shannon index and the number of OTUs for bacteria were negatively related to soil TN and DON in the 10–20 cm layer (Figure 2). Similar negative correlations for bacteria were observed between soil DOC and the Chao 1 index, the OTU number in the 10–20 cm layer, and between DOC and the Shannon index in the 20–40 cm layer. For fungi, in contrast, the Chao 1 index and the OTU number were positively correlated with soil DOC and AP in the 0–10 cm layer and with TC in the 20–40 cm layer; the Shannon index was negatively correlated with soil pH, soil moisture, TOC, and TN in the 10–20 cm layer (Figure 2).

### Composition of bacterial and fungal communities

**FIGURE 4:**
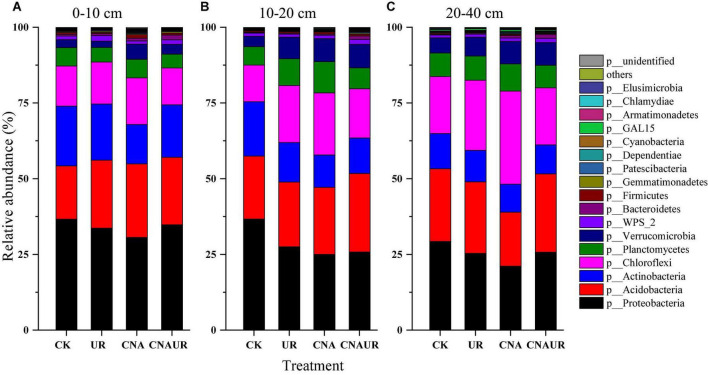
Relative abundance of soil bacterial community at the phyla level under treatments (treatments are described in Table 1) in three soil layers, 0–10 cm **(A)**, 10–20 cm **(B)**, 20–40 cm **(C)**. Those phyla that represent >0.1% of the bacterial community abundance are named, while those that represent <0.1% of the bacterial community abundance are referred to as “others.”

The following seven major bacterial phyla (relative abundance >1%) were identified across treatments and accounted for >94% of the bacterial community: Proteobacteria, Acidobacteria, Chloroflexi, Actinobacteria, Planctomycetes, Verrucomicrobia, and WPS_2 (Figure 4). The bacterial phyla in different soil layers were affected by CNA and UR (Supplementary Table 2). For example, CNA significantly increased the relative abundances of Verrucomicrobia and Chlamydiae in the 0–20 cm layer (Figures 4A,B) and Chloroflexi in the 10–40 cm layer (Figures 4B,C) but significantly decreased the relative abundances of Proteobacteria and Actinobacteria in the 10–20 cm layer (Figure 4B). A decrease in Proteobacteria relative abundance and an increase in Chloroflexi relative abundance were also observed for the UR treatment in the 10–40 cm soil layer (Figures 4B,C). In addition, the relative abundances of Chloroflexi and Planctomycetes in the 10–20 or 20–40 cm soil were affected by the statistical interaction between CNA and UR (Supplementary Table 2).

**FIGURE 5:**
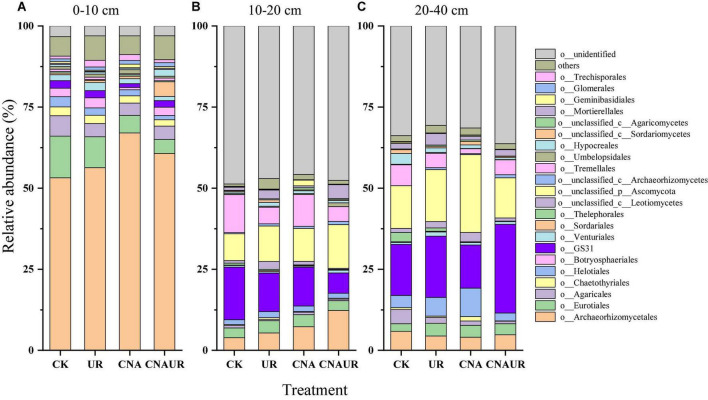
Relative abundance of soil fungal community at the order level under treatments (treatments are described in Table 1) in three soil layers, 0–10 cm **(A)**, 10–20 cm **(B)**, and 20–40 cm **(C)**. Those orders that represent >0.1% of the fungal community abundance are named, while those that represent <0.1% of the fungal community abundance are referred to as “others.”

The two most dominant phyla of fungi in all soil layers were Ascomycota (relative abundance = 36.1–87.5%) and Basidiomycota (5.7–15.3%); the less abundant phyla included Mortierellomycota (0.14–4.3%) and Mucoromycota (0.13–1.3%) (Supplementary Figure 1). The phylum Glomeromycota (0.85–1.30%) was only found in the 0–10 cm soil layer (Supplementary Figure 1A). Among the detected sequences, there were 45.8–48.7% and 29.0–36.3% of sequences unidentified in the 10–20 and 20–40 cm soil layer, respectively (Supplementary Figures 1B,C), although good coverage of sequences was in the range of 0.98–1.0 in these soil layers. Archaeorhizomycetales, Eurotiales, and Agaricales were the three most dominant orders of fungi in the 0–10 cm soil layer (Figure 5A). However, GS31, Tremellales, and Archaeorhizomycetales including unclassified p_Ascomycota were the dominant fungi in the 10–40 cm soil layer (Figures 5A,B). Moreover, CNA significantly decreased the relative abundances of order Archaeorhizomycetales, Eurotiales, Helotiales, and Venturiales, and the interaction of CNA and UR affected the relative abundance of Mortierellales in the 0–10 cm soil layer (Supplementary Table 4). In contrast, UR significantly increased the relative abundance of Mortierellales in the 10–20 cm layer (Supplementary Table 4) and decreased the relative abundance of Mortierellales and Chaetothyriales in the 20–40 cm layer (Supplementary Table 4). In addition, UR and the CNA × UR interaction significantly affected the relative abundance of the phylum Mortierellomycota in the 0–20 cm soil layer, while CNA significantly decreased the relative abundance of the phylum Mucoromycota in the 20–40 cm layer (Supplementary Table 3).

### Factors associated with bacterial and fungal community composition

**FIGURE 6:**
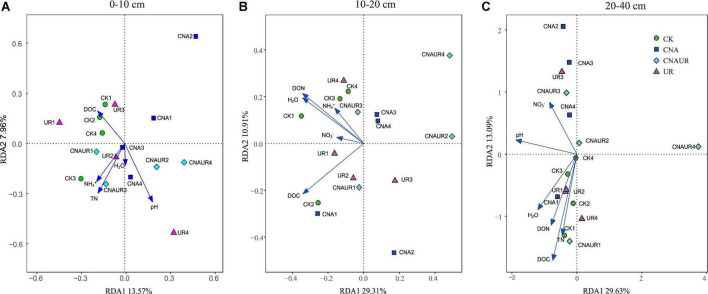
Redundancy analysis (RDA) of the relationship between bacterial community composition at the operational taxonomic (OTU) level and soil physicochemical properties across treatments in three soil layers, 0–10 cm **(A)**, 10–20 cm **(B)**, and 20–40 cm **(C)**.

**FIGURE 7:**
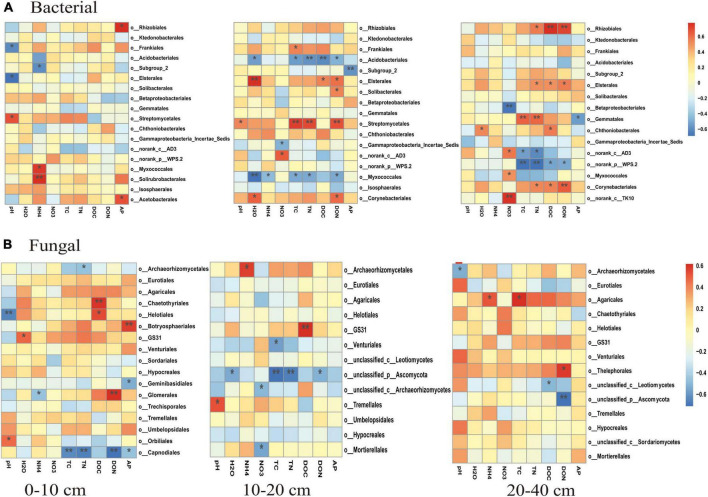
Correlations (as indicated by Pearson correlation coefficients) between soil properties and relative abundances of major bacterial taxa (>1.0%, **A–C**) and fungal taxa (>0.5%, **D–F**) at the order level in different soil layers. Blue indicates a negative correlation, and red indicates a positive correlation; the strength of the color indicates the strength of the correlation. * and ** indicate significant correlations at *p* < 0.05 and 0.01, respectively.

The PCoA analysis showed that the soil bacterial community structure in the 10–20 cm layer was significantly shifted by CNA and UR treatment (Supplementary Figure 2B, PERMANOVA test, *R*^2^ = 0.31, *p* = 0.013). In particular, the bacterial community composition was different with the CNA treatment than with the CK treatment. RDA analysis revealed that the first two RDA axes explained 16.1% of the variance in the bacterial community and that soil DON (*F* = 3.9, *p* = 0.008) followed by soil moisture and DOC were the most important factors associated with bacterial community composition in the 10–20 cm layer (Figure 6). However, soil TN (*F* = 2.3, *p* = 0.011) had the largest effect on the bacterial community in the 0–10 cm layer, and soil pH (*F* = 5.5, *p* = 0.038) and DOC (*F* = 4.3, *p* = 0.002) were the two important factors to bacterial community composition in the 20–40 cm soil layer (Figures 6A,C). Together, the RDA 1 and RDA 2 axes accounted for 40.22% of the variance in the 0–10 cm layer and 42.7% of the variance in the 20–40 cm layers. The SEM analysis showed that soil pH, DOC, and DON explained 29.00% of variance in the bacterial community (Supplementary Figure 3). In addition, the relative abundances were negatively correlated with pH for the orders Frankiales and Elsterales but were positively correlated with pH for the order Streptomycetales. Positive correlations were observed between NH_4_^+^ and Myxococcales and Solirubrobacteriales and between AP and Rhizobiales and Acetobacterales in the 0–10 cm layer (Figure 7). In the 10–20 cm layer, Elsterales and Corynebacteriales were positively correlated with soil moisture and DON; Streptomycetales were positively correlated with TC, TN, and DON; and Acidobacteriales and Myxococcales were negatively correlated with all soil parameters except NO_3_^–^ and AP. In the 20–40 cm layer, Rhizobiales, Elsterales, and Corynebacteriales were positively correlated with DOC and DON, and Gemmatales were positively correlated with TC and TN but were negatively correlated with AP (Figure 7B). Negative correlations were also found between Betaproteobacteriales and NO_3_^–^ and between norank taxa of phyla WPS.2 and class AD3 and TOC and TN.

**FIGURE 8:**
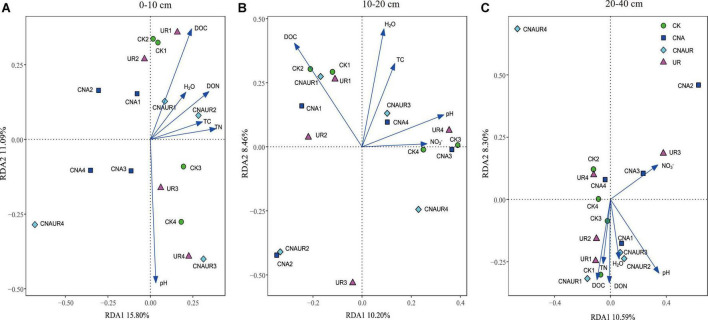
Redundancy analysis (RDA) of the relationship between fungal community composition at the operational taxonomic (OTU) level and soil physicochemical properties across treatments in three soil layers, 0–10 cm **(A)**, 10–20 cm **(B)**, and 20–40 cm **(C)**.

Unlike the bacterial community, fungal community composition significantly differed among treatments in the 0–10 cm soil layer (Supplementary Figure 4A, PERMANOVA test, *R*^2^ = 0.24, *p* = 0.034). In this layer, the fungal community composition was significantly associated with soil DOC (*p* = 0.002) and pH (*p* = 0.004), and in the RDA, the two axes (RDA 1 and RDA 2) were explained for 26.89% of the variation (Figure 8). The SEM analysis showed that soil pH, DOC, and DON explained 47.00% of variance in the bacterial community (Supplementary Figure 3). Moreover, the relative abundance of Helotiales was negatively correlated with pH but was positively correlated with DOC. Negative correlations were also observed between the relative abundance of Archaeorhizomycetales and TN, between the relative abundance of Geminibasidiales and AP, and between the relative abundance of Capnodiales and TOC, TN, and DON (Figure 7E). In contrast, the relative abundances of Botryosphaeriales, GS31, Glomerales, and Orbiliales were positively correlated with AP, soil moisture, DON, and pH, respectively. Although the treatments did not change fungal community composition in the 10–40 cm layer (Supplementary Figure 4), soil DOC (*F* = 1.4, *p* = 0.024) and pH (*F* = 1.6, *p* = 0.008) were closely related to fungal community composition in the 10–20 and 20–40 cm layer, respectively (Figures 8A,C). In the 10–20 cm layer, the relative abundances of Archaeorhizomycetales, GS31, and Tremellales were positively correlated with NH_4_^+^, DOC, and pH, respectively, but the relative abundance of an unidentified phylum of Ascomycota was negatively correlated with TOC, TN, soil moisture, and DON. Negative correlations were also found between the relative abundances of Mortierellales and unclassified taxa of the phylum Archaeorhizomycetes and NO_3_^–^ and between the relative abundance of Venturiales and TOC. In the 20–40 cm layer, however, the relative abundances of Agaricales and Thelephorales were positively correlated with NH_4_^+^, TOC, DON, respectively, and the relative abundances of Archaeorhizomycetales, an unidentified phylum of Ascomycota, and an unidentified class of Leotiomycetes were negatively correlated with pH, DOC, and DON, respectively (Figure 7F).

## Discussion

### Effects of canopy N addition and understory removal on microbial abundance

In the present study, the abundance of bacteria but not of fungi was affected by CNA (Figure 1), which was partly consistent with our first hypothesis but was inconsistent with previous results obtained with understory N addition (Wang J. P. et al., 2021) and with CNA (Zhao et al., 2020). In mixed deciduous forests in China, N addition significantly decreased the abundance of bacteria and fungi in two studies (Shi et al., 2016; Wang J. P. et al., 2021) but increased the abundance of fungi in a third study (Zhao et al., 2020). These conflicting results may be due to differences in soil pH and nutrient availability. In general, N addition reduces soil pH, resulting in changes in soil microbial communities because pH greatly affects soil bacterial and fungal abundance in subtropical forest soils (Zhao et al., 2013; Wang et al., 2018). Wang J. P. et al. (2021) observed that a decrease in microbial abundance was associated with a decrease in soil pH after 6 years of N addition. Moreover, the increased N availability due to N deposition reduced the N limitation on the growth of soil fungi (Zhao et al., 2020). In the current study, soil pH did not significantly differ between CNA and CK treatments in all three soil layers. Two reasons may explain the short-term experimental period and the canopy interception. However, the CNA treatment significantly decreased the soil DON content in the 20–40 cm layer (Figure 1C; Table 1). Like the decrease in soil DON, bacterial abundance decreased mainly in the 20–40 cm layer. This finding was further supported by the significant decrease in MBC content in the CNA treatment.

Contrary to the effect of CNA, removing understory plants significantly increased bacterial abundance in the deep soil (Figure 1C), which partly supported our second hypothesis. Researchers previously reported that the removal of the understory increased the soil temperature and N supply and reduced the soil water content (Wang et al., 2014; Giuggiola et al., 2018; Fang et al., 2021), which are important factors affecting microbial growth (Kamble et al., 2013). For instance, studies in *Eucalyptus* plantations showed that understory removal reduced microbial biomass in the surface soil (Wu et al., 2011; Zhao et al., 2013). However, in the current study, soil moisture and available N content did not differ between the UR and CK treatments and were not correlated with bacterial or fungal abundance (Figure 2). Xiong et al. (2008) also found that understory removal did not greatly affect soil physical and chemical properties in a subtropical *Acacia mangium* plantation in China. In the current study, we speculate that understory removal may have increased the soil temperature and thereby increased litter decomposition in the deep soil, thereby increasing the supply of C for microbial growth; C is considered the most limiting nutrient for soil microorganisms (Demoling et al., 2007). Although soil temperature was not measured in this study, a significant increase in soil MBC content with the UR treatment was observed in the 10–20 cm layer. Moreover, the ratio of active soil C to total C in response to understory removal had been previously observed to increase with soil depth (Xi et al., 2021). We also found that CNA × UR interaction significantly influenced the bacterial abundance and MBC content in the 20–40 cm soil layer, suggesting that soil microbial biomass and especially bacterial biomass were more sensitive to the treatments in the deep soil than in the surface soil.

### Effects of canopy N addition and understory removal on microbial diversity

Previous studies have indicated that N addition increases, decreases, or does not affect microbial diversity (Allison et al., 2007; Freedman et al., 2015; Nie et al., 2018; Li P. et al., 2019; Wu et al., 2019; Wang J. P. et al., 2021). For instance, N addition significantly decreased soil microbial diversity in a northern hardwood forest (Freedman et al., 2015) and in subtropical forests (Li et al., 2016; Nie et al., 2018; Wu et al., 2019) but increased bacterial and fungal diversity in a natural subtropical forest (Wang J. P. et al., 2021). Another study reported that soil bacterial diversity did not respond to N addition in two tropical rainforests (Li P. et al., 2019). In the current study, CNA increased bacterial diversity in the 10–20 cm layer but reduced fungal diversity in the 0–10 cm layer (Figure 3D; Supplementary Table 2). This finding was contrary to our first hypothesis and was inconsistent with Zhao et al. (2020), who found that canopy addition of N increased soil fungal diversity. A possible explanation for the inconsistency is the difference in forest type, i.e., the current study was conducted in a coniferous forest, and the study of Zhao et al. (2020) was conducted in a deciduous forest. Previous reports suggested that soil microorganisms seem to be more strongly influenced by coniferous trees than by deciduous trees (White et al., 2005), and that the effect of N deposition on the soil microbial community could differ depending on tree species (Zechmeister-Boltenstern et al., 2011). Similarly, microbial properties were reported to be more sensitive to N addition in a subtropical broadleaf forest than in a temperate deciduous forest, and the difference was attributed to the differences in the soil buffering systems (Shi et al., 2016). Additional evidence that forest type helps explain why CNA increased soil fungal diversity in Zhao et al. (2020) but not in the current study is provided by the pH data. In Zhao et al. (2020), the soil pH of the deciduous forest was significantly decreased by 5 years of CNA, but the same duration and level of CNA did not significantly affect soil pH or other soil chemical properties in the current study (Table 1; Supplementary Table 1). Interestingly most negative correlations between the Shannon index, soil pH, NO_3_^–^ content, and TN content were observed in the 10–20 cm soil layer (Figure 2), suggesting that soil depth may be an important factor regulating microbial diversity in response to N addition. Overall, the current and previous results suggest that the responses of microbial diversity to CNA may depend on forest type and soil layer.

Contrary to our expectations, understory removal had minor effects on bacterial and fungal diversity (Figure 3; Supplementary Table 2). In previous studies, understory removal reduced the input of labile C into the oil (Chen et al., 2019), thereby affecting the microbial activity and community composition (Demoling et al., 2007; Wan et al., 2019). In the current study, however, understory removal did not alter the contents of soil DOC and available N (i.e., NH_4_^+^, NO_3_^–^, and DON). Other researchers also found that understory removal did not change the soil DOC content (Wu et al., 2011) or the hydrolyzable N content (Xiong et al., 2008). We infer that understory removal in the current study did not affect the availability of substrates for soil microorganisms. In addition, we observed that soil fungal diversity was significantly lower in the 20–40 cm soil layer with the CNAUR treatment than with the CNA treatment (Figure 3F) and that a strong statistical interaction was found between CNA and UR (Supplementary Table 2). A possible explanation is that removing understory plants after N addition reduced the competition for nutrients between roots and microorganisms in the deep soil and increased soil N availability. The increase in N availability could reduce fungal diversity (Cox et al., 2010). A trend for DON content supports that explanation to increase in the CNAUR treatment in the 20–40 cm soil layer (Table 1).

### Composition of the microbial community in response to canopy N addition and understory removal

There were significant differences in the microbial communities among treatments in the current study, especially in the surface and middle soil layers (Supplementary Figures 2, 3). Furthermore, RDA analysis suggested that soil DON was a key factor affecting the composition of the bacterial community in the 10–20 cm layer (Figure 6B), while soil pH and DOC were key factors affecting the composition of the fungal community in the 0–10 cm layer (Figure 8A). The findings are consistent with our third hypothesis and with the results of Kang et al. (2021), who found that changes in the composition of soil microbial communities were associated with different factors in different soil layers.

Contrary to our first hypothesis, short-term (5 years) CNA significantly influenced the composition of soil bacterial and fungal communities (Supplementary Figures 2, 3). This finding supported most studies of understory N addition (Freedman et al., 2015; Wang T. et al., 2021) but was inconsistent with Shi et al. (2016), who found that CNA did not change the composition of soil microbial communities in a subtropical or temperate forest. The difference in experimental duration might explain this inconsistency. Soil samples were collected after 2 years of CNA in Shi et al. (2016) but after 5 years of CNA in the current study. Zhao et al. (2020) recently found that the composition of the soil fungal community was altered after 5 years of CNA in the same site as that studied by Shi et al. (2016). Moreover, the response of microbial taxa to CNA differed among soil layers in the current study (Figures 4, 5; Supplementary Tables 2, 4). It is generally accepted that with increases in resource availability, the abundance of copiotrophic taxa (i.e., Proteobacteria, Actinobacteria, and Ascomycota) increases, whereas the abundance of oligotrophic taxa (e.g., Verrucomicrobia and Basidiomycota) decrease (Fierer et al., 2012; Ho et al., 2017; Wu et al., 2019; Zhao et al., 2020; Wang J. P. et al., 2021). Li et al. (2016) also found that experimental N deposition increased the abundance of copiotrophic soil microorganisms. In the current study, however, CNA caused a decrease in the abundance of Proteobacteria and Actinobacteria in the 10–20 cm layer, an increase in the abundance of Verrucomicrobia in the 0–20 cm layer, and no significant changes in the abundances of fungal phyla (Figure 4; Supplementary Figure 1; Supplementary Table 2). The differences might be explained by the relationships between soil factors and single taxa at a finer taxonomic scale than used in the current study because physiological traits can vary greatly within a single microbial phylum (Ho et al., 2017; Wang J. P. et al., 2021). The latter explanation was supported by our finding that some fungal taxa at the order level were especially sensitive to N addition in the current study, as evidenced by significant reductions in the relative abundances of Eurotiales, Helotiales, and Venturiales in the surface soil in response to the CNA treatment (Figure 5; Supplementary Figure 1; Supplementary Table 3). Our correlation heatmap further indicated that soil factors had different correlations with single microbial taxa at the order level in different soil layers (Figure 7). For example, the relative abundance of the order Rhizobiales, as a potential N fixer, was positively correlated with AP in the surface soil and with DON and DOC in the deeper soil (Figure 7). The latter finding was inconsistent with Wang J. P. et al. (2021), who found that the abundance of Rhizobiales was negatively correlated with soil NH_4_^+^ and NO_3_^–^. The CNA treatment resulted in a sharp decrease in AP content in the surface soil (Table 1). Together, these findings suggest that reducing the availability of P, CNA may inhibit the decomposition of organic matter in the surface soil and consequently cause the nutrient limits for microbes in middle and deep soils due to reducing the carbon source input.

Relative to CNA, understory removal had less effect on the composition of the soil microbial communities at the OTU level (Supplementary Figures 2, 3). This result was inconsistent with results obtained from a eucalyptus plantation (Wu et al., 2011; Zhao et al., 2013). The latter studies found that understory removal changed soil microbial communities (as indicated by the ratio of fungal biomass to bacterial biomass) and that the changes were associated with soil temperature and water content. In the present study, the small effect of understory removal on soil microbial communities was associated with the failure of understory removal to change soil physical and chemical properties (Table 1). The possible explanation is the difference in acquiring resources among microbial species (Coleman et al., 2002). Because single taxa of bacteria or fungi within orders but not phylum were found to have positive or negative correlations with soil parameters (Figure 7), another possible reason for the failure of understory removal to significantly affect soil microbial communities is that dominant understory species (*B. chungii* and *D. dichotoma*) represent relatively insufficient resources for soil bacteria and fungi. Studies in eucalyptus plantations indicate that the litter of *D. dichotoma* has a high ratio of lignin to nitrogen and a low decomposition rate (Zhao et al., 2011; Liu et al., 2012). We also found that the composition of the bacterial community was more affected by the statistical interaction of CNA and understory removal in the middle soil layer than in the surface or deeper soil layer (Supplementary Table 3). Overall, our results suggest that understory removal and its interaction with CNA had slight effects on the composition of the bacterial community in surface and middle soil layers. Additional studies are needed on the long-term effects of understory removal on the composition of bacterial and fungal communities in different soil layers.

## Conclusion

The current study demonstrated that bacterial abundance in the deep soil layer was decreased by CNA but increased by understory removal. Furthermore, the diversity and composition of soil bacterial and fungal communities were affected more by CNA than by understory removal, and the effects depended on the soil layer. In the surface soil layer, CNA increased bacterial diversity and altered bacterial community structure. In contrast, CNA decreased fungal diversity and changed fungal community composition in the middle soil layer. Soil pH, DOC, and DON were associated with the responses of bacterial and fungal communities to the treatments in the different soil layers. In addition, the bacterial community in the surface soil was also influenced by the statistical interaction between CNA and UR. Our results indicate that the soil bacterial community was more sensitive than the fungal community to CNA and understory removal in the short term.

## Data availability statement

The original contributions presented in this study are included in the article/Supplementary material, further inquiries can be directed to the corresponding authors.

## Author contributions

DX and JW performed the field experiments. DX and SJ performed the statistical analysis. DX wrote the first draft of the manuscript. All authors contributed to the conception and design of the study and manuscript revision, read, and approved the submitted version.

## Footnotes

1
http://sourceforge.net/projects/rdp-classifier/

2
http://www.arb-silva.de

3
http://unite.ut.ee/index.php
