# Supplementary material for: Soil bacterial community is more sensitive than fungal community to canopy nitrogen deposition and understory removal in a Chinese fir plantation
Source: Front Microbiol. 2022 Oct 12;13:1015936. doi: 10.3389/fmicb.2022.1015936 (PMC9597510; doi:10.3389/fmicb.2022.1015936)
Supplement: Supplementary file 1 [file Data_Sheet_1.docx]

Supplementary Material

# Supplementary Figures and Tables

## Supplementary Figures


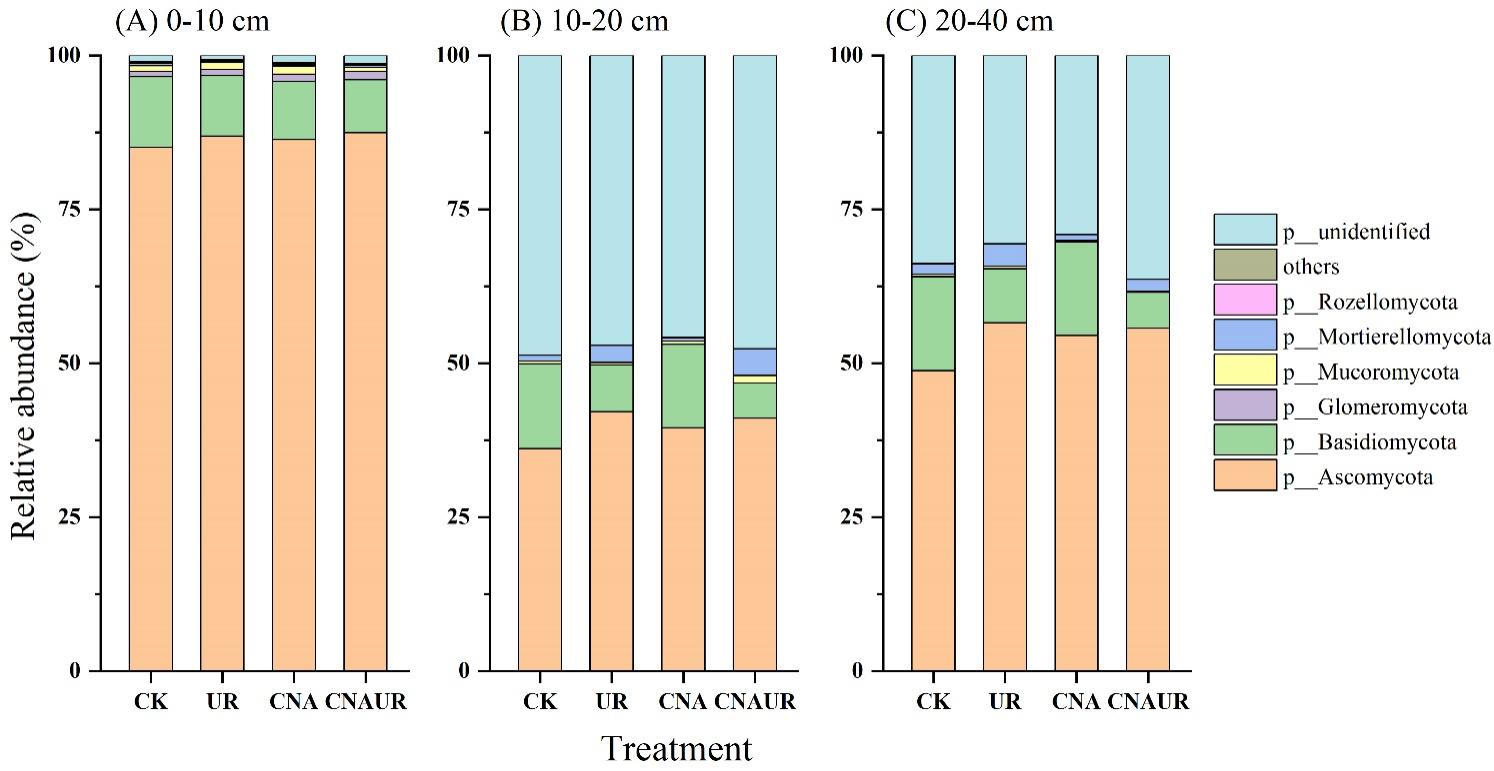


**Supplementary Figure S1.** Relative abundance of soil fungal community at the phyla level under treatments. Those phyla that represent > 0.1% of the fungal community abundance are named, while those that represent < 0.1% of the bacterial community abundance are referred to as “others”. CK: no N addition and no understory removal; UR: understory removal without N addition; CNA: canopy N addition (25 kg N ha^-1^yr^–1^) without understory removal; CNAUR: canopy N addition (25 kg N ha^-1^yr^–1^) plus understory removal.


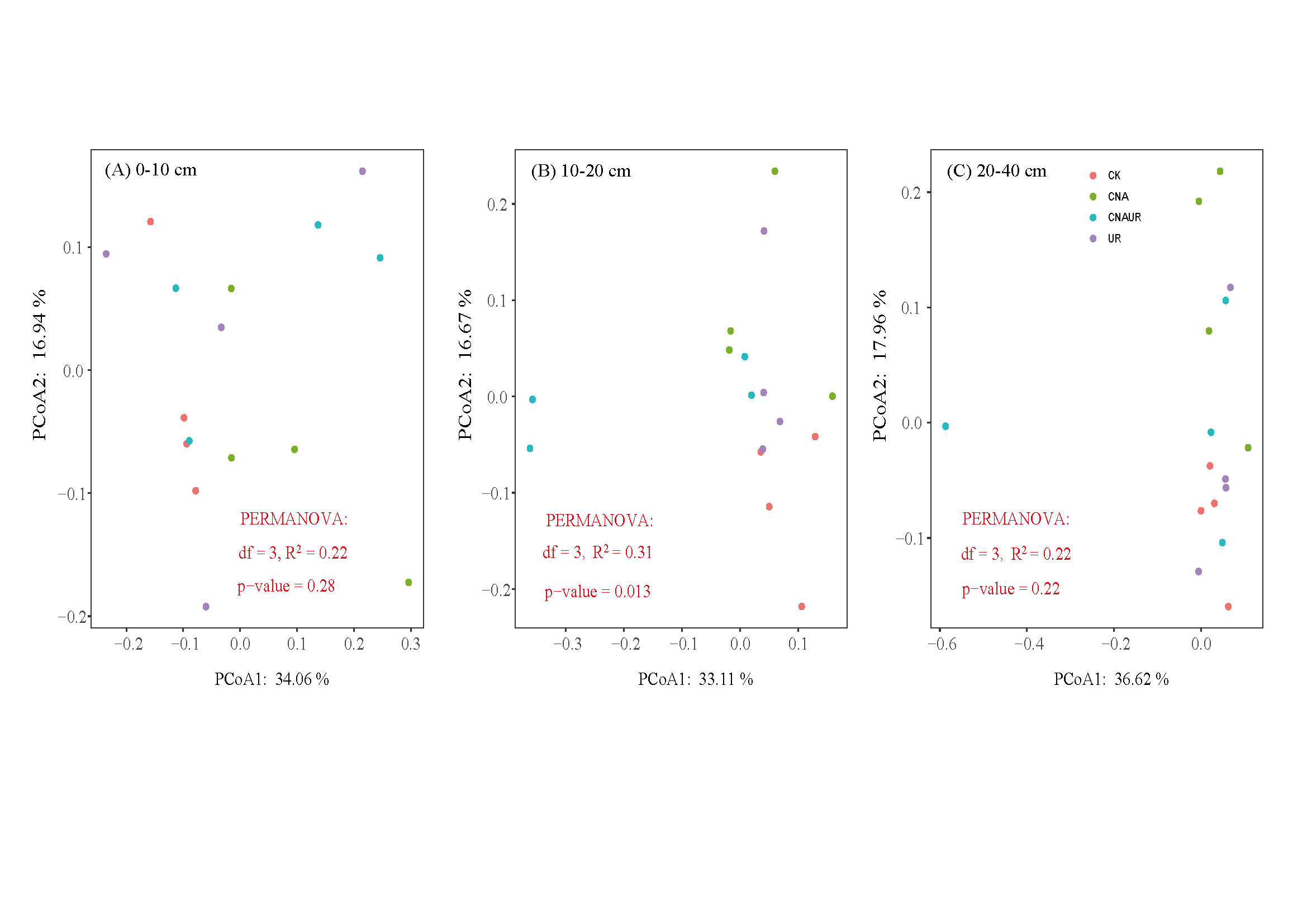


**Supplementary Figure S2.** Principle component analysis of bacterial (A-C) communities across treatments in different soil layers based on the operation taxonomic unit (OTU) tables. PERMANOVA test with adonis method was used to determine the compositional variation. CK: no N addition and no understory removal; UR: understory removal without N addition; CNA: canopy N addition (25 kg N ha^-1^yr^–1^) without understory removal; CNAUR: canopy N addition (25 kg N ha^-1^yr^–1^) plus understory removal.


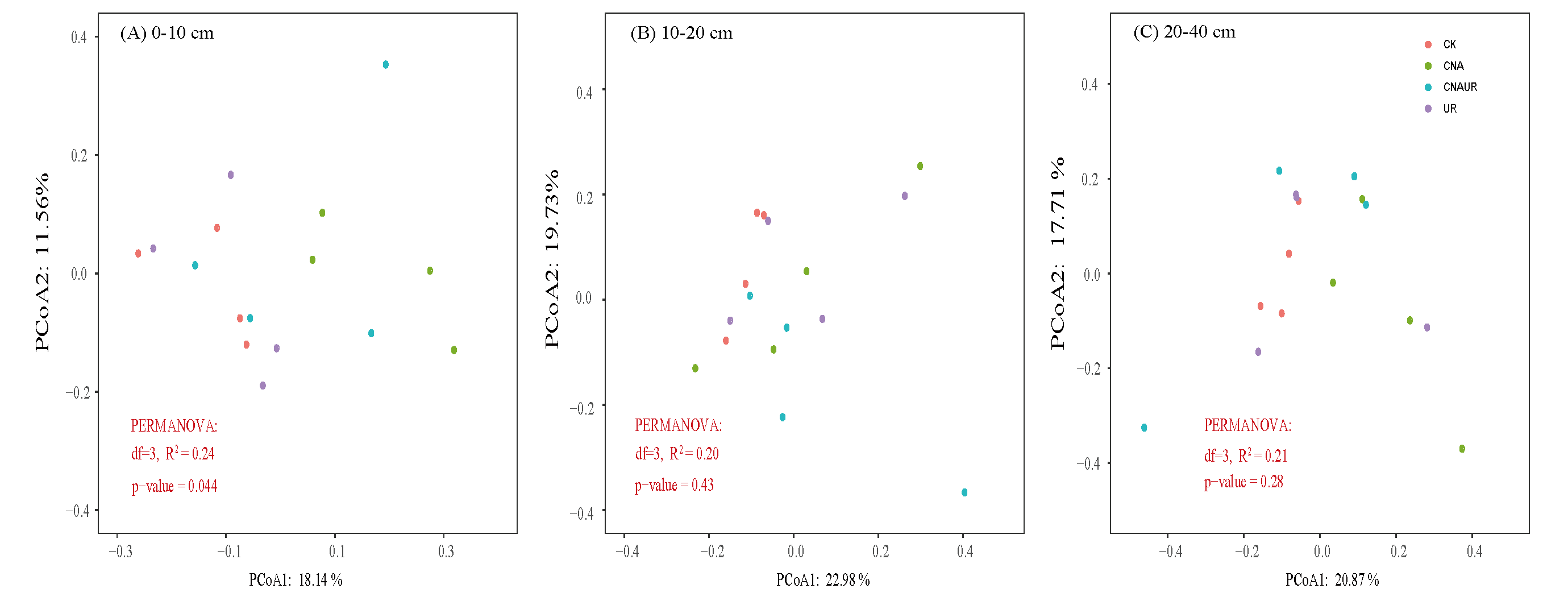


**Supplementary Figure S4.** Principle component analysis of fungal community compositions across treatments in the three soil layers (A-C), based on the operation taxonomic unit (OUT) tables. PERMANOVA test with adonis method was used to determine the compositional variation. CK: no N addition and no understory removal; UR: understory removal without N addition; CNA: canopy N addition (25 kg N ha^-1^yr^–1^) without understory removal; CNAUR: canopy N addition (25 kg N ha^-1^yr^–1^) plus understory removal.


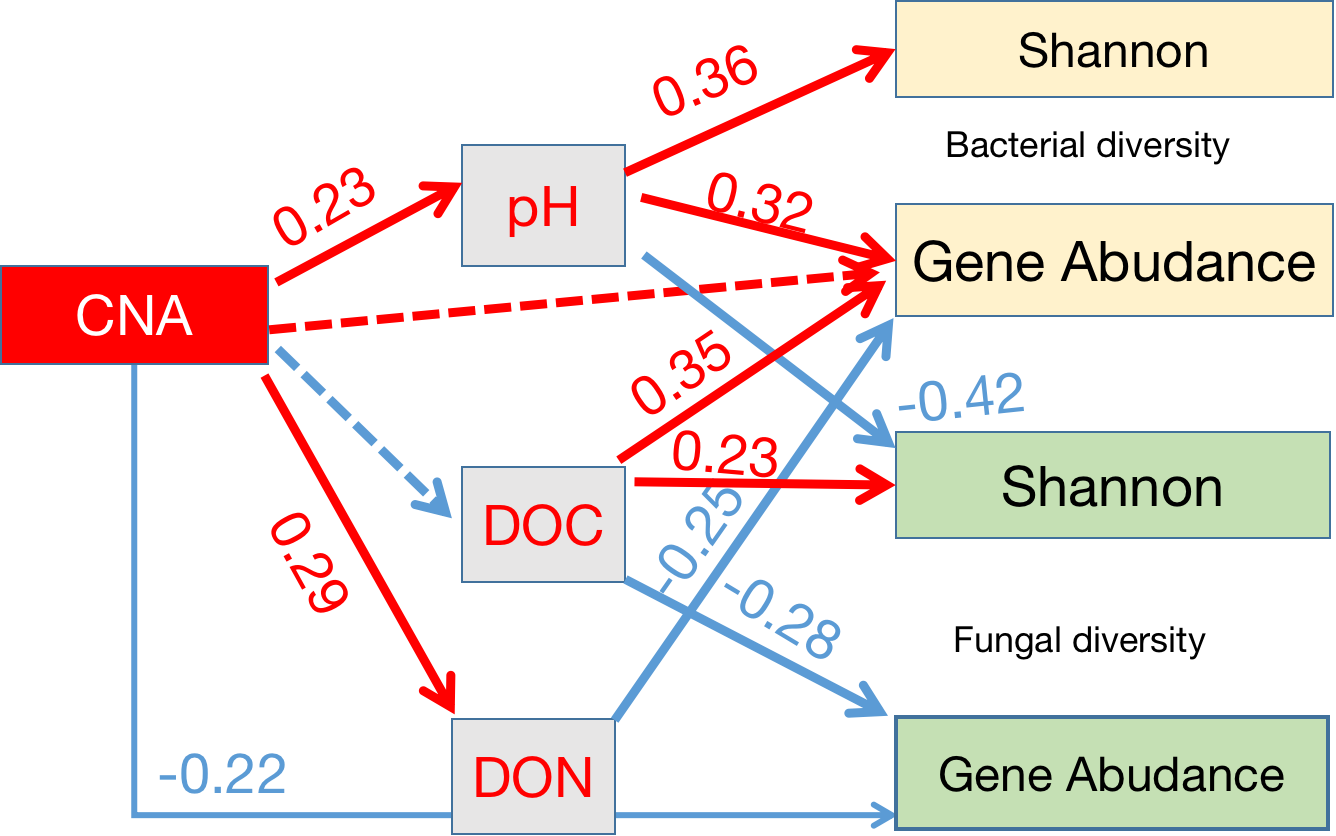


## Figure S3. Structure equation model (SEM) analysis of canopy nitrogen addition on soil microbial diversity via pathways of soil pH, DOC, and DON. Square boxes show variables included in the model. Solid and dashed arrows indicate significant and non-significant effects based on *P* < 0.05, respectively. Values associated with solid arrows indicate standardized path coefficients. The red and blue arrows indicate the positive and negative coefficients, respectively. Based on the model validation from the “piecewiseSEM” package, the global goodness-of-fit of this model is Fisher's C = 25.908; *P* = 0.102, and n=16.

## Supplementary Tables

**Table S1.** Effects of canopy N addition (CNA) and understory removal (UR) on soil physicochemical properties.

|  | 0-10 cm | | | | | |  | 10-20 cm | | | | | |  | 20-40 cm | | | | | |
| --- | --- | --- | --- | --- | --- | --- | --- | --- | --- | --- | --- | --- | --- | --- | --- | --- | --- | --- | --- | --- |
| Properties | CNA | | UR | | CNA×UR | |  | CNA | | UR | | CNA×UR | |  | CNA | | UR | | CNA×UR | |
|  | F | P | F | P | F | P |  | F | P | F | P | F | P |  | F | P | F | P | F | P |
| pH | 0.81 | 0.39 | 0.24 | 0.64 | 0.01 | 0.95 |  | 0.04 | 0.85 | 1.82 | 0.20 | 0.75 | 0.40 |  | 0.96 | 0.35 | 0.77 | 0.40 | 1.65 | 0.22 |
| H_2_O(%) | 0.08 | 0.79 | 0.05 | 0.84 | 0.27 | 0.61 |  | 0.92 | 0.36 | 0.07 | 0.80 | 0.44 | 0.52 |  | 1.85 | 0.20 | 0.02 | 0.89 | 2.39 | 0.15 |
| TOC (g kg^-1^) | 0.01 | 0.94 | 2.18 | 0.17 | 1.44 | 0.25 |  | 1.46 | 0.25 | 0.17 | 0.68 | 0.14 | 0.71 |  | 0.03 | 0.88 | 0.01 | 0.92 | 0.04 | 0.84 |
| TN (g kg^-1^) | 0.06 | 0.81 | 2.14 | 0.17 | 1.81 | 0.20 |  | 1.42 | 0.26 | 0.14 | 0.72 | 0.03 | 0.88 |  | 0.01 | 0.95 | 0.15 | 0.70 | 0.02 | 0.89 |
| NH_4_^+^ (mg kg^-1^) | 1.07 | 0.32 | 0.28 | 0.61 | 2.23 | 0.16 |  | 0.07 | 0.79 | 1.09 | 0.32 | 0.01 | 0.94 |  | 0.04 | 0.85 | 0.18 | 0.68 | 0.43 | 0.52 |
| NO_3_^-^ (mg kg^-1^) | 2.26 | 0.16 | 0.83 | 0.38 | 0.97 | 0.34 |  | 1.80 | 0.20 | 1.56 | 0.24 | 0.05 | 0.82 |  | 0.63 | 0.44 | 2.73 | 0.12 | 1.54 | 0.24 |
| DON (mg kg^-1^) | 0.16 | 0.69 | 0.01 | 0.99 | 0.06 | 0.82 |  | 1.73 | 0.21 | 0.10 | 0.75 | 0.93 | 0.35 |  | 2.19 | 0.17 | 0.73 | 0.41 | 4.51 | 0.06* |
| DOC (mg kg^-1^) | 0.23 | 0.64 | 0.03 | 0.88 | 0.01 | 0.99 |  | 0.06 | 0.81 | 0.13 | 0.73 | 0.28 | 0.61 |  | 0.78 | 0.39 | 0.01 | 0.93 | 0.07 | 0.80 |
| AP (mg kg^-1^) | 1.17 | 0.30 | 0.35 | 0.56 | 4.05 | 0.07* |  | 0.03 | 0.88 | 0.002 | 0.97 | 3.42 | 0.09* |  | 0.01 | 0.99 | 1.00 | 0.34 | 1.21 | 0.29 |
| MBC (mg kg^-1^) | 1.26 | 0.28 | 1.50 | 0.24 | 0.03 | 0.89 |  | 0.16 | 0.69 | 4.04 | 0.07* | 6.34 | **0.03** |  | 0.001 | 0.98 | 2.01 | 0.18 | 6.45 | **0.03** |
| MBN (mg kg^-1^) | 0.78 | 0.40 | 0.02 | 0.88 | 1.39 | 0.26 |  | 1.13 | 0.25 | 2.15 | 0.17 | 0.42 | 0.53 |  | 1.20 | 0.30 | 4.47 | 0.06 | 0.23 | 0.64 |

Notes: * represents significant difference at the 0.1 level; ** and bold value represents significant difference at the 0.05 level;.

**Table S2.** Effects of canopy N addition (CN) and understory removal (UR) on bacterial diversity and relative abundance of community composition at phyla level.

|  | 0-10 cm | | | | |  |  | 10-20 cm | | | | | |  |  | | 20-40 cm | | | | | | |
| --- | --- | --- | --- | --- | --- | --- | --- | --- | --- | --- | --- | --- | --- | --- | --- | --- | --- | --- | --- | --- | --- | --- | --- |
| Bacterial | UR |  | CNA |  | CNA×UR | |  | UR |  | CNA |  | CNA×UR | | |  | UR | |  | CNA |  | CNA×UR | |  |
|  | F | P | F | P | F | P |  | F | P | F | P | F | P | |  | F | | P | F | P | F | P |  |
| observed OTUs | 1.33 | 0.27 | 1.53 | 0.24 | 0.83 | 0.38 |  | 2.78 | 0.12 | 1.48 | 0.25 | 0.35 | 0.57 | |  | 0.47 | | 0.51 | 0.09 | 0.77 | 0.21 | 0.66 |  |
| Chao1 index | 0.15 | 0.71 | 0.62 | 0.45 | 1.93 | 0.19 |  | 0.69 | 0.42 | 0.07 | 0.8 | 0.73 | 0.41 | |  | 0.59 | | 0.46 | 1.03 | 0.33 | 0.99 | 0.34 |  |
| Shannon index | 0.82 | 0.38 | 3.17 | 0.10 | 0.86 | 0.37 |  | 6.85 | **0.02** | 6.25 | **0.03** | 0.09 | 0.78 | |  | 0.03 | | 0.88 | 0.27 | 0.62 | 0.05 | 0.83 |  |
| 16s rRNA gene copy (copies g^-1^ soil) | 0.46 | 0.51 | 0.005 | 0.95 | 0.01 | 0.91 |  | 0.02 | 0.89 | 2.22 | **0.161** | 0.45 | 0.52 | |  | 9.36 | | **0.01** | 9.29 | **0.01** | 10.57 | **0.007** |  |
| p__Proteobacteria | 0.04 | 0.86 | 0.60 | 0.46 | 1.27 | 0.28 |  | 2.72 | 0.13 | 6.82 | **0.02** | 3.74 | 0.08 | |  | 0.02 | | 0.88 | 2.87 | 0.12 | 3.49 | 0.09 |  |
| p__Acidobacteria | 0.13 | 0.73 | 0.63 | 0.44 | 0.69 | 0.42 |  | 0.99 | 0.34 | 1.65 | 0.22 | 0.52 | 0.48 | |  | 1.89 | | 0.19 | 0.51 | 0.49 | 2.32 | 0.15 |  |
| p__Actinobacteria | 0.34 | 0.57 | 2.06 | 0.18 | 0.96 | 0.35 |  | 1.27 | 0.28 | 6.12 | **0.03** | 2.84 | 0.12 | |  | 0.16 | | 0.70 | 2.14 | 0.17 | 0.53 | 0.48 |  |
| p__Chloroflexi | 0.35 | 0.57 | 0.01 | 0.93 | 0.66 | 0.43 |  | 0.25 | 0.63 | 1.40 | 0.26 | 4.94 | **0.046** | |  | 1.12 | | 0.31 | 1.13 | 0.31 | 5.16 | **0.04** |  |
| p__Planctomycetes | 1.09 | 0.32 | 0.002 | 0.95 | 0.02 | 0.90 |  | 0.10 | 0.76 | 1.24 | 0.29 | 7.52 | **0.02** | |  | 0.38 | | 0.55 | 0.09 | 0.77 | 0.61 | 0.45 |  |
| p__Verrucomicrobia | 6.30 | **0.03** | 14.29 | **0.002** | 1.56 | 0.24 |  | 2.58 | 0.13 | 3.72 | 0.08 | 2.18 | 0.17 | |  | 0.16 | | 0.69 | 1.32 | 0.27 | 0.15 | 0.71 |  |
| p__WPS_2 | 3.21 | 0.10 | 0.70 | 0.42 | 0.15 | 0.70 |  | 1.83 | 0.20 | 3.57 | 0.08 | 1.94 | 0.19 | |  | 0.43 | | 0.52 | 1.34 | 0.27 | 0.27 | 0.62 |  |
| p__Bacteroidetes | 1.32 | 0.27 | 2.72 | 0.13 | 0.69 | 0.42 |  | 2.46 | 0.14 | 3.58 | 0.08 | 1.86 | 0.20 | |  | 0.53 | | 0.48 | 4.72 | 0.05 | 0.29 | 0.60 |  |
| p__Firmicutes | 1.12 | 0.31 | 1.38 | 0.26 | 0.47 | 0.51 |  | 0.03 | 0.86 | 0.80 | 0.39 | 0.18 | 0.68 | |  | 1.34 | | 0.27 | 0.002 | 0.96 | 0.001 | 0.98 |  |
| p__Gemmatimonadetes | 2.81 | 0.12 | 0.74 | 0.41 | 2.27 | 0.16 |  | 3.74 | 0.08 | 1.37 | 0.27 | 4.60 | 0.05 | |  | 0.02 | | 0.89 | 0.48 | 0.50 | 0.002 | 0.99 |  |
| p__Patescibacteria | 1.11 | 0.31 | 0.19 | 0.67 | 1.48 | 0.25 |  | 2.82 | 0.12 | 1.53 | 0.24 | 1.25 | 0.29 | |  | 0.10 | | 0.76 | 6.53 | **0.03** | 0.22 | 0.65 |  |
| p__Dependentiae | 2.67 | 0.13 | 0.01 | 0.94 | 0.67 | 0.43 |  | 0.71 | 0.42 | 0.42 | 0.53 | 0.85 | 0.38 | |  | 0.08 | | 0.79 | 0.01 | 0.92 | 2.23 | 0.16 |  |
| p__Cyanobacteria | 3.38 | 0.09 | 1.55 | 0.24 | 0.06 | 0.82 |  | 1.29 | 0.28 | 1.84 | 0.20 | 0.71 | 0.42 | |  | 0.20 | | 0.67 | 0.11 | 0.74 | 1.90 | 0.19 |  |
| p__GAL15 | 0.61 | 0.45 | 1.05 | 0.33 | 0.23 | 0.64 |  | 2.70 | 0.13 | 0.18 | 0.68 | 0.55 | 0.47 | |  | 0.12 | | 0.74 | 0.001 | 0.97 | 0.59 | 0.46 |  |
| p__Armatimonadetes | 0.01 | 1.00 | 3.84 | 0.07 | 0.33 | 0.58 |  | 3.55 | 0.08 | 1.86 | 0.20 | 1.59 | 0.23 | |  | 0.38 | | 0.55 | 1.36 | 0.27 | 0.24 | 0.63 |  |
| p__Chlamydiae | 8.24 | **0.01** | 1.92 | 0.19 | 4.67 | 0.05 |  | 0.27 | 0.61 | 5.56 | **0.04** | 6.34 | **0.03** | |  | 3.24 | | 0.10 | 0.002 | 0.95 | 0.07 | 0.80 |  |
| p__Elusimicrobia | 9.96 | **0.01** | 3.66 | 0.08 | 6.43 | **0.03** |  | 1.03 | 0.33 | 0.28 | 0.61 | 0.10 | 0.76 | |  | 0.002 | | 0.97 | 0.06 | 0.81 | 0.42 | 0.53 |  |

Notes: Bold value represents significant difference at the 0.05 level.

**Table S3.** Effects of canopy N addition (CNA) and understory removal (UR) on fungal diversity and relative abundances of community composition at phyla level.

| Fungi | 0-10 cm | | | | | |  | 10-20 cm | | | | | |  | | 20-40 cm | | | | | | |
| --- | --- | --- | --- | --- | --- | --- | --- | --- | --- | --- | --- | --- | --- | --- | --- | --- | --- | --- | --- | --- | --- | --- |
|  | UR |  | CNA |  | UR × CNA | |  | UR |  | CNA |  | UR ×CNA | |  | UR | |  | CNA |  | UR ×CNA | |  |
|  | F | P | F | P | F | P |  | F | P | F | P | F | P |  | F | | P | F | P | F | P |  |
| observed OTUs | 2.94 | 0.11 | 1.29 | 0.28 | 0.95 | 0.35 |  | 0.01 | 0.94 | 1.23 | 0.29 | 2.71 | 0.13 |  | 0.18 | | 0.68 | 2.72 | 0.13 | 0.82 | 0.38 |  |
| Chao 1 index | 0.68 | 0.43 | 4.98 | **0.04** | 0.05 | 0.83 |  | 0.29 | 0.60 | 0.61 | 0.45 | 1.86 | 0.20 |  | 0.01 | | 0.91 | 2.51 | 0.14 | 1.14 | 0.31 |  |
| Shannon index | 4.24 | 0.06 | 8.81 | **0.01** | 1.80 | 0.20 |  | 2.02 | 0.18 | 0.15 | 0.70 | 1.45 | 0.25 |  | 3.93 | | 0.07 | 0.39 | 0.54 | 5.54 | **0.04** |  |
| ITS gene copy (copies g^-1^ soil) | 0.08 | 0.78 | 0.84 | 0.36 | 0.001 | 0.99 |  | 0.36 | 0.07 | 0.38 | 0.06 | 0.97 | 0.10 |  | 0.79 | | 0.40 | 0.19 | 0.67 | 0.07 | 0.80 |  |
| p__Ascomycota | 0.53 | 0.48 | 0.20 | 0.66 | 0.03 | 0.87 |  | 0.42 | 0.53 | 0.04 | 0.85 | 0.14 | 0.71 |  | 0.33 | | 0.58 | 0.10 | 0.76 | 0.18 | 0.68 |  |
| p__Basidiomycota | 0.42 | 0.53 | 0.69 | 0.42 | 0.04 | 0.84 |  | 1.33 | 0.27 | 0.03 | 0.87 | 0.02 | 0.89 |  | 1.54 | | 0.24 | 0.06 | 0.82 | 0.05 | 0.82 |  |
| p__Glomeromycota | 0.31 | 0.59 | 1.04 | 0.33 | 0.002 | 0.97 |  | 1.99 | 0.18 | 0.83 | 0.38 | 0.75 | 0.40 |  | 3.13 | | 0.10 | 0.61 | 0.45 | 0.61 | 0.45 |  |
| p__Mucoromycota | 0.14 | 0.71 | 0.001 | 0.98 | 0.28 | 0.60 |  | 1.66 | 0.22 | 2.88 | 0.12 | 2.26 | 0.16 |  | 0.07 | | 0.80 | 6.24 | **0.03** | 0.15 | 0.71 |  |
| p__Mortierellomycota | 0.57 | 0.46 | 0.29 | 0.60 | 10.14 | **0.01** |  | 18.43 | **0.001** | 0.80 | 0.39 | 2.08 | 0.18 |  | 3.53 | | 0.09 | 2.19 | 0.17 | 0.39 | 0.55 |  |
| p__Rozellomycota | 0.03 | 0.88 | 0.14 | 0.71 | 0.08 | 0.78 |  | 0.56 | 0.47 | 0.31 | 0.59 | 0.14 | 0.72 |  | 0.03 | | 0.87 | 0.57 | 0.47 | 1.04 | 0.33 |  |

Notes: Bold value represents significant difference at the 0.05 level.

| Fungi order | 0-10 cm | | | | | |  | 10-20 cm | | | | | |  | | 20-40 cm | | | | | | |
| --- | --- | --- | --- | --- | --- | --- | --- | --- | --- | --- | --- | --- | --- | --- | --- | --- | --- | --- | --- | --- | --- | --- |
|  | UR | | CNA | | UR × CNA | |  | UR | | CNA | | UR × CNA | |  | | UR | | CNA | | UR × CNA | | |
|  | F | P | F | P | F | P |  | F | P | F | P | F | P |  | F | | P | F | P | F | P |  |
| o__Archaeorhizomycetales | 0.14 | 0.71 | 4.53 | **0.05** | 1.22 | 0.29 |  | 1.61 | 0.23 | 3.98 | 0.07 | 0.48 | 0.50 |  | 0.007 | | 0.93 | 0.48 | 0.50 | 0.85 | 0.37 |  |
| o__Eurotiales | 1.62 | 0.23 | 14.17 | **0.003** | 0.45 | 0.51 |  | 0.001 | 0.97 | 0.001 | 0.99 | 0.96 | 0.35 |  | 0.311 | | 0.59 | 0.27 | 0.61 | 1.10 | 0.32 |  |
| o__Agaricales | 0.59 | 0.46 | 0.83 | 0.38 | 0.94 | 0.35 |  | 0.80 | 0.39 | 0.001 | 0.97 | 0.024 | 0.88 |  | 1.01 | | 0.33 | 2.60 | 0.13 | 0.70 | 0.42 |  |
| o__Helotiales | 2.72 | 0.12 | 6.59 | **0.025** | 0.13 | 0.73 |  | 0.032 | 0.86 | 0.005 | 0.94 | 0.32 | 0.58 |  | 0.59 | | 0.46 | 0.001 | 0.97 | 3.57 | 0.08 |  |
| o__GS31 | 0.33 | 0.58 | 0.95 | 0.35 | 0.44 | 0.52 |  | 1.43 | 0.25 | 1.36 | 0.26 | 0.025 | 0.88 |  | 2.66 | | 0.13 | 0.08 | 0.79 | 1.25 | 0.28 |  |
| o__Venturiales | 0.46 | 0.51 | 4.81 | **0.049** | 1.64 | 0.22 |  | 0.63 | 0.44 | 0.23 | 0.64 | 0.84 | 0.38 |  | 1.28 | | 0.28 | 0.13 | 0.73 | 0.48 | 0.50 |  |
| o__Sordariales | 0.97 | 0.34 | 1.05 | 0.32 | 0.79 | 0.39 |  | 0.62 | 0.45 | 0.004 | 0.95 | 1.12 | 0.31 |  | 0.13 | | 0.72 | 3.14 | 0.10 | 0.38 | 0.55 |  |
| o__Thelephorales | 1.58 | 0.23 | 0.02 | 0.90 | 0.17 | 0.69 |  | 0.28 | 0.60 | 3.78 | 0.08 | 0.13 | 0.72 |  | 0.85 | | 0.38 | 3.29 | 0.09 | 0.73 | 0.41 |  |
| o__Tremellales | 1.67 | 0.22 | 0.85 | 0.37 | 1.49 | 0.25 |  | 0.98 | 0.34 | 0.044 | 0.84 | 0.01 | 0.92 |  | 0.084 | | 0.78 | 1.06 | 0.32 | 1.177 | 0.30 |  |
| o__Umbelopsidales | 0.12 | 0.73 | 0.003 | 0.96 | 0.36 | 0.56 |  | 3.09 | 0.10 | 2.82 | 0.119 | 3.43 | 0.09 |  | 0.075 | | 0.79 | 2.02 | 0.18 | 0.001 | 1.00 |  |
| o__Hypocreales | 1.95 | 0.19 | 1.17 | 0.30 | 2.16 | 0.17 |  | 0.78 | 0.39 | 0.038 | 0.85 | 2.10 | 0.17 |  | 0.84 | | 0.38 | 1.22 | 0.29 | 0.24 | 0.63 |  |
| o__Chaetothyriales | 0.11 | 0.74 | 0.82 | 0.38 | 0.027 | 0.87 |  | 2.98 | 0.11 | 3.04 | 0.11 | 1.09 | 0.32 |  | 6.79 | | **0.023** | 0.61 | 0.45 | 3.21 | 0.098 |  |
| o__Mortierellales | 0.57 | 0.46 | 0.29 | 0.60 | 10.18 | **0.008** |  | 17.48 | **0.001** | 0.85 | 0.37 | 2.14 | 0.17 |  | 3.52 | | 0.085 | 2.185 | 0.16 | 0.39 | 0.55 |  |

**Table S4.** Effects of canopy N addition (CNA) and understory removal (UR) on fungal community composition at order level.

Notes: Bold value represents significant difference at the 0.05 level.
